# Comparing the effects of argon plasma coagulation and interferon therapy in patients with vaginal intraepithelial neoplasia: a single-center retrospective study

**DOI:** 10.1007/s00404-024-07477-3

**Published:** 2024-04-29

**Authors:** Yuan Gao, Weixin Chu, Lin Hou, Junlan Cheng, Guyue Zhong, Baoguo Xia, Li Guo

**Affiliations:** 1 Department of Gynecology, Qingdao Municipal Hospital, Qingdao, Shandong China; 2 Department of Biochemistry and Molecular Biology, Basic Medical College, Qingdao University, Qingdao, Shandong China; 3 Department of Gynecology, The Second Affiliated Hospital of Shandong First Medical University, Taian, Shandong China; 4 Department of Gynecology, Ansteel Group General Hospital, Anshan, Liaoning China; 5 Department of Gynecology, Qingdao Municipal Hospital, Qingdao University, Qingdao, Shandong China

**Keywords:** Vaginal intraepithelial neoplasia, Argon plasma coagulation, Interferon, Efficacy, Safety

## Abstract

**Purpose:**

This study aimed to evaluate the clinical efficacy and safety of argon plasma coagulation (APC) therapy and interferon therapy in patients with grade I and II vaginal intraepithelial neoplasia (VaIN).

**Methods:**

A total of 112 patients with VaIN were diagnosed via colposcopy-induced biopsy and classified into the APC group (*n* = 77) and interferon group (*n* = 35). Clinical data including age, grade, symptoms, historical or concomitant neoplasia of the lower genital tract, indications for hysterectomy, pregnancy history, cytology, human papillomavirus (HPV) subtype, treatment modalities, and clinical outcomes were analyzed, retrospectively. Complications and clinical outcomes were assessed at 6- and 12-month follow-ups.

**Results:**

There was no significant difference in the HPV clearance rate between the APC (53.42%) and interferon (33.33%) groups at 6 months after treatment. However, the 12-month follow-up of the APC group showed a significantly higher HPV clearance rate as compared to the interferon group (87.67% vs. 51.52%, *P* < 0.05). The APC group exhibited a significantly higher cure rate (79.22% vs. 40.0%) and lower persistence rate (12.99% vs. 37.14%) than the interferon group (*P* < 0.05). Adverse reaction analysis revealed that the primary reaction in the APC group was vaginal drainage, in contrast to the increased vaginal discharge in the interferon group; though the difference was significant (68.83% vs. 28.57%, *P* < 0.05), no serious complications were observed.

**Conclusions:**

Treatment with APC is a safe and more effective procedure against VaIN I and II, compared to interferon. APC may serve as a viable alternative to other physiotherapies.

## What does this study add to the clinical work

**Table:** 

Treatment with APC is a safe and more effective procedure against VaIN I and II compared to interferon. APC may serve as a viable alternative to other physiotherapies.	

## Introduction

Vaginal intraepithelial neoplasia (VaIN) is defined as atypical hyperplasia of different levels limited to the vaginal intraepithelial tissue. VaIN is precancerous lesion that could potentially lead to vaginal carcinoma. In 1952, Graham and Meigs reported on cases of vaginal carcinoma in situ during follow-up after hysterectomy for cervical carcinoma, and first proposed the concept of vaginal intraepithelial neoplasia [1]. The incidence of VaIN is 0.2–2 per 100,000 women/year [2, 3], accounts for only 1.0% of cervical intraepithelial neoplasia (CIN) cases, and 0.4%–1.0% of cases of premalignant lesions in the lower genital tract of females [4, 5]. However, with the widespread application of Thinprep cytology test (TCT), human papillomavirus (HPV) detection and colposcopy in cervical cancer screening, and increased disease awareness, the prevalence of VaIN has increased steadily in recent years [6]. The clinical manifestations of VaIN are atypical and few patients present with increased vaginal secretion or contact bleeding. The classical three-step diagnostic model for CIN includes cytological analysis and/or HPV–colposcopy–histopathology is recommended for VaIN diagnosis. Histopathological diagnosis guided by colposcopy is considered the gold standard [7].

In accordance with the WHO classification system for tumors in female reproductive organs, VaIN is classified into low-grade squamous intraepithelial lesions (LSIL, VaIN I) and high-grade squamous intraepithelial lesions (HSIL, VaIN II, VaIN III) [8]. According to the consensus statement, VaIN management procedures vary based on the grade of the lesion: patients with VaIN I could undergo follow-up because of the low risk of progression and high potential for spontaneous regression. Conversely, as VaIN II–III have premalignant potential, patients with such lesions should be treated promptly [2]. However, not all VaIN I patients can reverse, and some lesions may persist or even progress [9]. Patients with recurrent or widespread vaginal LSIL or those with a history of CIN/cervical cancer tend to be treated actively [10]. The general treatment options for VaIN include drug therapy (imiquimod, 5-fluorouracil, interferon, estrogen), physiotherapy (CO_2_ laser, cryotherapy, photodynamic therapy, electrocoagulation), surgical excision (loop electrosurgical excision procedure, cavitational ultrasonic surgical aspiration, partial/total vaginectomy, laparoscopic upper vaginal resection), and brachytherapy [11–13]. Strategies should be stratified according to risk and personalized according to the lesion grade, patient’s general conditions, age, previous medical history, disease extension, recurrence risk, acceptance of treatment methods, physicians’ experience, and other factors.

Interferon (IFN) has antiviral, immunoregulatory, and antitumor effects, and plays a critical role in the treatment of HPV clearance, chronic hepatitis B/C, and multiple sclerosis [14]. IFN is widely used for treating HPV-related cervical lesions in China [15]. Despite the safety profile has been deemed acceptable, information on their efficacy for VaIN patients is limited. The CO_2_ laser is used widely in the clinic and has the most treatment experience among physiotherapy, but might lead to local adhesion and vaginal scarring [16]. Photodynamic therapy is another common treatment method; however, it requires the use of photosensitizers and often needs to be performed several times [6]. Therefore, a more safer and user-friendly treatment needs to be found for VaIN patients. Argon plasma coagulation (APC) is a non-contact ablative technique through igniting argon gas into a plasma to cauterize and devitalize vascular tissues to achieve hemostasis or debulking tumors, such as endometriosis or ovarian tumor implants [17, 18]. The argon plasma beam can automatically avoid the solidification zone and flow to the insufficient solidification zone, thereby significantly reducing the risk of over solidification [19, 20]. In 2012, the FDA determined that the plasma energy system was substantially equivalent to the CO_2_ laser system and could thus be used for similar purposes [21]. APC has been applied for treating gastric low-grade intraepithelial neoplasia, gallbladder cancer, colon polyps, Barrett’s esophagus, and vulval intraepithelial neoplasia [22–26].

However, to date, only one study has evaluated the complications and recurrence rates associated with the use of APC for treating vulvovaginal dysplasia, which included only 16 cases of vaginal HSIL [21]. In the current study, we performed a retrospective analysis of clinical data obtained from VaIN patients treated with APC or interferon, to compare their treatment outcomes and evaluate the efficacy of argon plasma coagulation treatment.

## Materials and methods

### Study participants

This was a retrospective study that analyzed clinical data from 112 patients treated at the Inpatient Department of Gynecology and Obstetrics at Qingdao Municipal Hospital from January 2018 to November 2022. All patients underwent colposcopy biopsy prior to treatment and were diagnosed with VaIN I or VaIN II. Seventy-seven patients received APC treatment, and 35 received interferon therapy. This study was conducted after receiving approval from the Hospital Ethics Committee, and all patients signed an informed consent form before undergoing treatment. The inclusion criteria were as follows: (1) histopathological diagnosis of VaIN guided by colposcopy; (2) patients undergoing a minimum of 12 months of follow-up. The exclusion criteria were as follows: (1) vaginal cancer; (2) acute reproductive tract infection; (3) history of chemoradiotherapy; (4) severe cardiac, liver and renal insufficiency, or immune disease; (5) pregnancy or lactation; (6) patients without routine follow-up. Clinical data including the age, grade, symptoms, historical or concomitant neoplasia of the lower genital tract, indications for hysterectomy, pregnancy history, TCT results, HPV subtype, treatment modalities, and clinical outcomes, were collected.

### Procedure

#### Argon plasma coagulation

All treatments were administered in a day surgery setting using an APC therapeutic instrument (Erbe Elektromedizin, Germany, VIO300D) at an output power of 45 W with an argon gas flow of 3.2 L/min. Each patient underwent one-time therapy at 3–7 days after menstruation in a conscious state. Intravenous anesthesia was administered to post-hysterectomy patients to enhance the exposure of retracted vaginal folds and vault corners. Lugol’s iodine was applied at the vagina to outline the non-stained disease area. Ablation was performed from the lesion margin at a distance of 0.3–0.5 cm, with the probe positioned 2–3 mm away from the lesion, until the tissue surface was covered by a yellow and coagulated layer without bleeding. Patients with multifocal disease were treated at the same time. If there was no bleeding or other complication, the patient was discharged on the same day. The procedure was performed by the same accredited specialist colposcopist to ensure a constant APC strategy.

#### Interferon

An interferon α2b capsule (800,000 IU) was inserted into the vaginal fornix in a lithotomy position every night. All patients were treated once a day for 3 months, except during menstruation. Patients were prohibited from having sexual intercourse during treatment and were advised to use condoms after treatment.

#### Cytology, HPV genotype, and colposcopy

Cytology test was performed using the Tinprep 2000 (TCT). HPV genotype analysis was performed by the HPV 21 Genotyping Assay (Hybribio, Guangdong, China), which tests for 15 high-risk HPV types (HPV 16, 18, 31, 33, 35, 39, 45, 51, 52, 53, 56, 58, 59, 66, 68) and 6 low-risk HPV types (HPV 6, 11, 42, 43, 44, 81). Patients with abnormal TCT and/or HPV genotype results were assessed via 3MLLED electronic colposcopy (Leisegang, German). The inclusion criteria for colposcopy were as follows: (1) the TCT revealing atypical squamous cells of undetermined significance (ASCUS) with high-risk HPV infection; (2) HPV 16 and/or HPV 18 infection; (3) the TCT showing LSIL or HSIL, or atypical squamous cells, without excluding high-grade squamous intraepithelial lesions (ASC-H); (4) persistent infection with high-risk HPV subtype after treatment.

### Follow-up and efficacy evaluation

Adverse events, including fever, pain, vaginal bleeding or drainage, pruritus, adhesion, injury of bladder or rectum, or other findings were documented during the checkup and patients were provided targeted treatment if necessary. The first follow-up after treatment was scheduled 1 month later, along with a pelvic examination. Subsequent follow-ups were scheduled every 6 months for 2 years, then annually. At 6 and 12 months, TCT and HPV genotyping were performed and, if indicated, colposcopy and biopsy were performed by an experienced gynecologic oncologist. VaIN diagnosis were confirmed by two independent pathologists.

Clinical outcomes were classified into four types: cure, persistence, recurrence, and progression. Cure: no indication for colposcopy according to the screening results, negative colposcopy examination, or negative biopsy at the 6-month follow-up. Persistence: the VaIN grade remained unchanged or decreased by biopsy at the 6-month follow-up. Recurrence: the initial disease was cured at the 6-month follow-up, followed by subsequent recurrence at the 12-month follow-up, as confirmed via biopsy. Progression: biopsy-proven higher grade or invasive cancer at the 6-month follow-up. The HPV clearance rate refers to the proportion of patients with negative conversion of HPV after treatment in patients who were positive for HPV before treatment.

### Statistical analysis

Statistical analysis was conducted using SPSS25.0 statistics software (SPSS, Inc., Chicago, USA). Continuous variables were expressed as the mean and standard deviation values. Categorical variables were expressed as percentage values and analyzed using the Chi-square test or Fisher’s exact test. A *P* value less than 0.05 was considered statistically significant.

## Results

### Clinical characteristics of patients

A total of 112 women were included, of whom 77 patients received APC therapy, and 35 patients received interferon treatment. Patient characteristics between the two groups showed no statistical significance (Table 1). Patients in the study were aged between 21–76 years (mean: 45.71 ± 13.82). Most patients (77.6%) were asymptomatic at diagnosis. Among all the 112 patients, 28 (25%) had a prior history of cervical neoplasia. Fifteen patients (13.3%) underwent hysterectomy before receiving a VaIN diagnosis. The most common indication for hysterectomy was cervical cancer (73.3%), followed by high-grade CIN (26.7%). Among the 97 patients who did not undergo hysterectomy, 56 (57.7%) had concurrent cervical neoplasia. The general data of patients (including age, gravidity, parity, histopathology, symptoms) were similar between the two groups (*P* > 0.05).

**Table 1:** Patient characteristics

Characteristic	APC (*n* = 77)	Interferon (*n* = 35)	*P*-value
Age			
< 50	47	16	0.13
≥ 50	30	19	
Gravidity			
≤ 1	29	10	0.349
≥ 2	48	25	
Parity			
≤ 1	58	27	0.835
≥ 2	19	8	
Previous cervical neoplasia history			
None	61	23	0.301
CIN	10	7	
Cervical cancer	6	5	
Previous hysterectomy			
Yes	9	6	0.550
No	68	29	
Hysterectomy indication (*n* = 15)			
CIN/CIS	3	1	0.604
Cervical cancer	6	5	
Concurrent cervical neoplasia			
Yes	43	13	0.067
No	34	22	
Histopathology			
VaIN I	48	25	0.349
VaIN II	29	10	
Symptoms			
No	59	28	0.721
Abnormal vaginal bleeding	10	5	
Abnormal vaginal discharge	8	2	

*VaIN* vaginal intraepithelial neoplasia, *CIN* cervical intraepithelial neoplasia

The results of cytological and HPV genotype analysis are shown in Table 2. Among the 77 patients in the APC group, there were 22, 20, 25, 7, and 3 cases of NILM, ASCUS, LSIL, HSIL, and ASC-H, respectively; 73 patients were HPV positive and 36 had multiple HPV infections. In the control group, 33 patients were HPV positive and 15 had multiple HPV infections; the TCT results showed that 16, 6, 11, 1, and 1 patients were with conditions NILM, ASCUS, LSIL, HSIL and ASC-H, respectively. There was no significant difference between the two groups (*P* > 0.05).

**Table 2:** Cytology test results and HPV infection status for patients

Characteristic	APC (*n* = 77)	Interferon (*n* = 35)	*P* value
Cytology test results			
NILM	22	16	0.369
ASCUS	20	6	
LSIL	25	11	
HSIL	7	1	
ASC-H	3	1	
HPV status			
HPV ( +)	73	33	1.000
HPV (−)	4	2	
HPV infection			
Single HPV infection	37	18	0.713
Multiple HPV infections	36	15	
HPV genotypes			
Negative	4	2	0.645
HPV 16	29	10	
HPV 18	6	5	
Others	41	20	

*NILM* negative for intraepithelial lesion or malignancy, *ASC-US* atypical squamous cells of undetermined significance, *LSIL* low-grade squamous intraepithelial lesions, *HSIL* high-grade squamous intraepithelial lesions, *ASC-H* atypical squamous cells, *HSIL* cannot be excluded; *HPV* human papillomavirus

### Baseline data in correlation with the VaIN grade

At the time of diagnosis, 73 (65.2%) and 39 (34.8%) patients were diagnosed with VaIN I and VaIN II, respectively. The differences in previous history of cervical neoplasia or hysterectomy, and cytology results were not significant among the two grades. ASCUS was the most common finding in VaIN I cases, while squamous intraepithelial lesion (SIL) occurred most commonly in females with VaIN II. However, the proportion of HSIL increases gradually (from 5.48% to 28.21%) with the increase grade of VaIN (Table 3).

**Table 3:** Clinicopathologic characteristics, cytology test results, and HPV infection status in correlation with VaIN grade

Characteristic	VaIN I (*n* = 73)	VaIN II (*n* = 39)	*P*-value
Previous cervical neoplasia history			
None	56 (76.71%)	27 (69.23%)	0.626
CIN	10 (13.7%)	8 (20.51%)	
Cervical cancer	7 (9.59%)	4 (10.26%)	
Previous hysterectomy			
Yes	8 (10.96%)	7 (17.95%)	0.301
No	65 (89.04%)	32 (82.05%)	
Concurrent cervical neoplasia			
No	38 (52.05%)	18 (46.15%)	0.136
CIN I	22 (30.14%)	7 (17.95%)	
CIN II	9 (12.33%)	8 (20.51%)	
CIN III	4 (5.48%)	6 (15.38%)	
Cytology test results			
NILM	26 (35.62%)	12 (30.77%)	0.766
ASCUS	16 (21.92%)	10 (25.64%)	
LSIL	25 (34.25%)	11 (28.21%)	
HSIL	4 (5.48%)	11 (28.21%)	
ASC-H	2 (2.74%)	2 (5.13%)	
HPV status			
HPV ( −)	4 (5.48%)	2 (5.13%)	1.000
HPV ( +)	69 (94.52%)	37 (94.87%)	
HPV infection			
Single HPV infection	34 (49.28)	21 (56.76%)	0.462
Multiple HPV infections	35 (50.72)	16 (43.24%)	
HPV genotypes			
Negative	4 (5.33%)	2 (5.0%)	0.012
HPV 16	17 (22.67%)	21 (52.5%)	
HPV 18	8 (10.67%)	2 (5.0%)	
Others	46 (61.33%)	15 (37.5%)	

All patients were tested to determine their HPV genotypes. There was a significant statistical difference between the HPV genotype and VaIN grade. In patients with VaIN I, other types of HPV occurred more commonly than HPV 16 (61.33% vs. 22.67%). However, the proportion was reversed in patients with VaIN II (37.5% vs. 52.5%) (*P* < 0.001; Table 3). Among the various genotypes of HPV, the top five most common genotypes were HPV 16 (36.79%), HPV 58 (25.47%), HPV 59 (13.21%), HPV 53 (12.26%), HPV 18 (10.38%), and HPV 56 (10.38%) (Table 4).

**Table 4:** HPV genotypes in VaIN

HPV infection (*n* = 106)	*N*^a^	Percentage
High-risk HPV		
HPV 16	39	36.79%
HPV 18	11	10.38%
HPV 31	9	8.49%
HPV 33	9	8.49%
HPV 35	3	2.83%
HPV 39	4	3.77%
HPV 45	0	0%
HPV 51	6	5.66%
HPV 52	9	8.49%
HPV 53	13	12.26%
HPV 56	11	10.38%
HPV 58	27	25.47%
HPV 59	14	13.21%
HPV 66	5	4.72%
HPV 68	5	4.72%
Low-risk HPV		
HPV 6	4	3.77%
HPV 11	2	1.89%
HPV 42	2	1.89%
HPV 43	3	2.83%
HPV 44	1	0.94%
HPV 81	6	5.66%

^a^Because of the presence of multiple HPV infections, the total number of cases in the table is more than 106

### Clearance rate of HPV infection

At 6 months after treatment, there was no significant difference in the HPV clearance rate between the APC (53.42%, 39/73) and interferon (33.33%, 11/33) groups. However, the group that underwent a 12-month follow-up after APC exhibited a significantly higher HPV clearance rate than the interferon group (87.67%, (64/73) vs. 51.52%, (17/33), *P* < 0.05) (Table 5).

**Table 5:** Human papillomavirus clearance rate

	APC (*n* = 73)	Interferon (*n* = 33)	*P* value
HPV clearance rate at 6-month			
HPV( −)	53.42% (39/73)	33.33% (11/33)	0.055
HPV( +)	46.58% (34/73)	66.67% (22/33)	
HPV clearance rate at 12-month			
HPV( −)	87.67% (64/73)	51.52% (17/33)	< 0.001
HPV( +)	12.33% (9/73)	48.48% (16/33)	

### Clinical efficacy

**Fig. 1:**
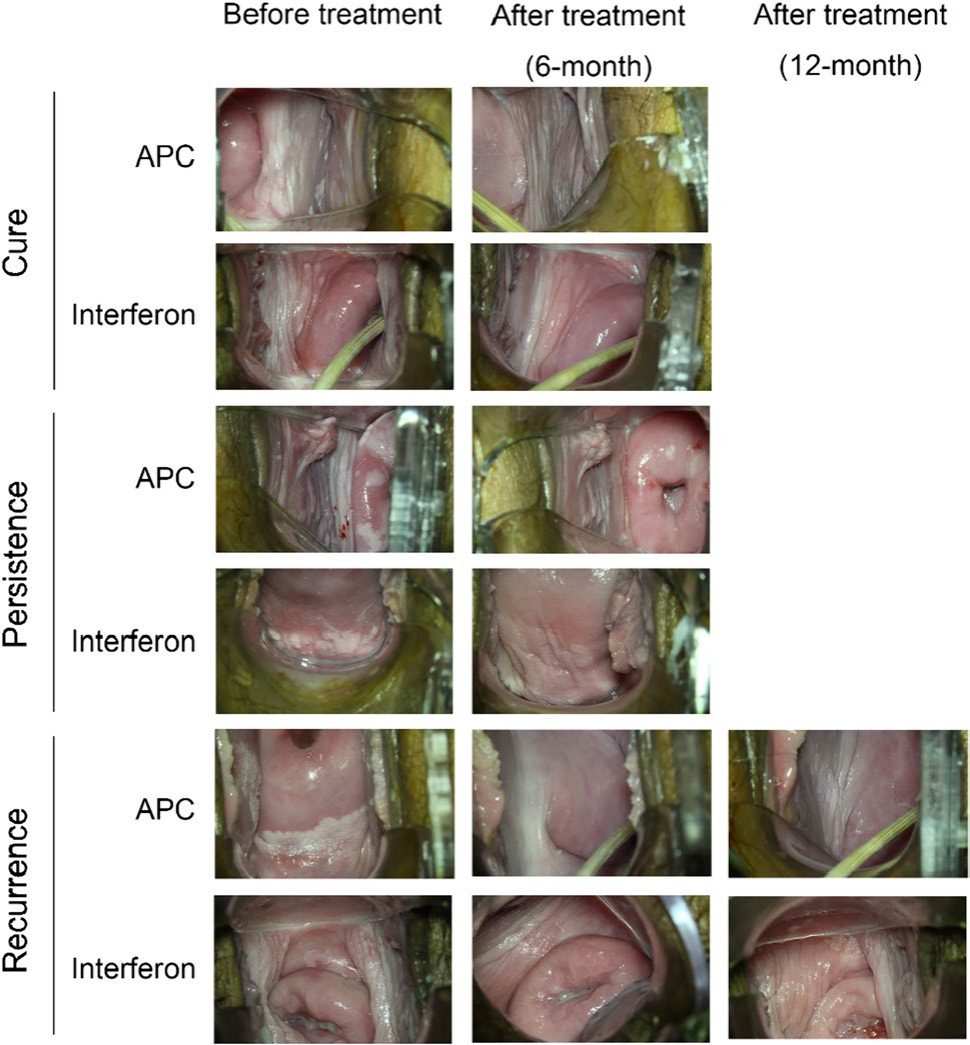
Colposcopy images before and after treatment of APC and interferon therapy groups

Based on the 6-month and 12-month follow-up data, among 77 patients in the APC group, 61 (79.22%), 10 (12.99%), 5 (6.49%), and 1 (1.3%) patients experienced cure, persistence, recurrence, and progression, respectively. The corresponding proportion in the interferon group was 40.0% (14/35), 37.14% (13/35), 14.29% (5/35), and 8.57% (3/35), respectively. The APC group exhibited a significantly higher cure rate and lower persistence rate than the interferon group (*P* < 0.05) (Table 6) (Fig. 1).

**Table 6:** Evaluation of the clinical efficacy

Outcome	APC (*n* = 77)	Interferon (*n* = 35)	*P* value
Cure			
Yes	61 (79.22%)	14 (40.0%)	< 0.001
No	16 (20.78%)	21 (60.0%)	
Persistence			
Yes	10 (12.99%)	13 (37.14%)	0.003
No	67 (87.01%)	22 (62.86%)	
Recurrence			
Yes	5 (6.49%)	5 (14.29%)	0.281
No	72 (93.51%)	28 (85.71%)	
Progression			
Yes	1 (1.3%)	3 (8.57%)	0.09
No	76 (98.7%)	32 (91.43%)

### Adverse reactions

To determine the safety of APC therapy, we analyzed the rate and type of adverse reactions observed in our patient cohort. The main adverse reaction observed in the APC group was vaginal drainage (53/77), and increased vaginal discharge was observed in the interferon group (10/35), and the differences were significant (*P* < 0.05). The symptom of vaginal drainage appeared at 7–14 days after treatment and relieved after approximately 30–40 days. Notably, during the 1-month follow-up, eight patients in the APC group with multifocal lesions developed adhesions. However, these adhesions were loose and could be separated easily using cotton swabs. The vaginal mucosa was smooth and elastic, without obvious scarring and contracture during subsequent long-term follow-ups. Three patients in the APC group experienced vaginal bleeding, which required the use of gauze to apply pressure in the vagina. Of note, those patients underwent concurrent excision and ablation for CIN, and blood appeared from the cervical wound. Overall, most patients experienced no side effects or mild adverse reactions, such as burning or pruritus (Table 7).

**Table 7:** Adverse reactions after treatment

Symptom	APC (*n* = 77)	Interferon (*n* = 35)	*P* value
Vaginal drainage or increased discharge	53 (68.83%)	10 (28.57%)	< 0.001
Burning sensation	3 (3.9%)	5 (14.29%)	0.105
Pruritus of the genital	7 (9.09%)	6 (17.14%)	0.222
Abnormal bleeding	3 (3.9%)	0 (0.0%)	0.551
Vaginal adhesion	8 (10.39%)	0 (0.0%)	0.055

## Discussion

In recent years, the diagnosis and treatment of VaINs have received increased attention. Due to the absence of typical clinical manifestations, the incidence of VaIN has been relatively underestimated. In our study, most patients (77.6%) were asymptomatic at diagnosis, with only a few patients showing abnormal vaginal bleeding or discharge. Age is a high risk factor for VaIN, with a notable association observed between the grade of VaIN and age [27]. Postmenopausal women showed a 2.09 times higher incidence compared to premenopausal women, due to decreasing estrogen levels, reduced local vaginal resistance, and increasing susceptibility to HPV infection [28]. A retrospective study of 3229 VaIN patients reported that the mean age of the patients with VaIN I, II, and III was 47.1, 47.1, and 49.9 years, respectively [27]. The results of our study showed that the mean age was 45.7 years, which is consistent with previous studies. Notably, 10.7% (12/112) of the participants were under the age of 25. VaIN present a trend of youth along with the increasing rate of HPV infection, highlighting the attention for clinicians not to ignore young patients.

HPV infection has been proven to be the main etiologic factor for neoplasia in the lower genital tract of females. Mengyin et al. [27] revealed that the HPV positivity rates of patients with VaIN I, II, and III were 89.0%, 92.5%, and 96.8%, respectively, and the rate of multiple infections was lowest in VaIN III. However, Chao et al. suggested that the lowest rate of multiple infections could be observed in patients with VaIN II [29]. Our results showed the same findings regarding the prevalence rate of HPV, which was 94.52% in VaIN I, and 94.87% in VaIN II. Compared with the multiple HPV infections (50.72%) of VAIN I, single infection (56.76%) was the main pattern of VaIN II. These studies suggested that there may be no correlation between the HPV infection pattern and VaIN grade. The predominant HPV genotype in VaIN patients was HPV 16, followed by 58, 59, 53, 18, and 56 in our study. Infections with other types (61.33%) were more common in patients with VaIN I, and HPV16 (52.5%) occurred most commonly in those with VaIN II, which was slightly in contrast with the findings of previous studies [27, 30]. This might be attributed to variations in different regions, nations, or samples. In addition to the above risk factors, the following reasons might also increase the incidence of VaIN: a history of CIN or cervical cancer (CC), a previous hysterectomy for HPV-related disease, or concomitant cervical neoplasia [31, 32]. In the present study, 57.7% of patients had concurrent cervical neoplasia, and 25% had a prior history of cervical neoplasia. Therefore, all patients scheduled for hysterectomy due to CIN or CC should undergo adequate colposcopy examination before surgery to prevent the possibility of overlooking a VaIN diagnosis. During colposcopy, meticulous observation of the vaginal folds, especially the fornix, is of great importance. Long cotton swabs or long flat tweezers can be used if necessary.

Until now, the management of VaIN has remained controversial, and there is no unanimous agreement on the best method. As one of the first-line therapies against CIN, interferon can up-regulate the chemotaxis of macrophages and natural killer cells in tissues infected with virus and further modulate the immune system [33]. APC is an ablative technique that yields results by expelling ionized argon gas onto the target mucosal surface, thereby transferring high-frequency electrical energy to tissues, utilizing thermal effects to deactivate and dry the tissue and cause coagulation and necrosis [19]. The first use of argon plasma energy in gynecological surgery was reported by Madhuri et al. in 2010, to treat ovarian cancer, ovarian cyst, peritoneal cancer, endometriosis, and myoma [34]. Until now, the non-invasive physical plasma (NIPP) treatment using a next-generation and non-thermally electrosurgical argon plasma device was widely used in precancerous and cancerous lesions, such as CIN [35]. However, only one retrospective cohort study had been conducted to evaluate the effectiveness of APC in patients with vulvovaginal HSIL. After a median follow-up period of 29.3 months for 41 patients treated for vulvar (*n* = 25) or vaginal (*n* = 16) HSIL, the recurrence rates (33.3%) and complication rates (4.8%) in the plasma ablation group were similar to the laser ablation group [21]. However, the effect of APC on VaIN has not been evaluated extensively because of the small sample size and only patients with HSIL were included.

In this study, we compared the HPV clearance rates, clinical efficacies, and adverse reactions between patients treated with the interferon and APC therapies. The negative conversion rates of HPV in the APC and interferon groups were 53.42% and 33.33%, respectively, after 6 months, showing no statistical significance (*P* > 0.05); however, after 12 months, the values were 87.67% and 51.52%, respectively, and differences were statistically significant, indicating the long-term effect of APC. In the interferon group, lesions disappeared at 6 months in 40% of patients, but recurred at 12 months in 14.29% of the patients. According to Gonçalo et al., approximately 44%–81% of all types of VaIN lesions (*N* = 468) can regress spontaneously, and the rate was 68.8% in LSIL [11]. Our study showed that the efficacy of interferon therapy was even lower than the expectant management reported by Gonçalo, suggesting that interferon might not be effective for the treatment of VaIN. However, further studies with larger samples and longer follow-up periods are required to confirm this viewpoint. The APC group had a significantly higher cure rate (79.22%) and lower persistence rate (12.99%) than the interferon group (*P* < 0.05), with the recurrence rate being only 6.49%. The results were similar to those observed upon treatment with other ablation methods [36]. This suggests that APC is more effective than interferon, and can be considered as an alternative physiotherapy strategy for VaIN patients. As a non-thermal APC application, NIPP has been demonstrated to affect cellular processes through gene methylation and phosphorylation of H2AX, p53, and p53-binding protein 1, ultimately inducing tissue-preserving responses in all mucosal tissue layers of CIN [37].Weiss et al. observed an 86.2% rate of full remission, a 3.4% rate of partial remission, and a twofold reduction in high-risk HPV infections in CIN1/2 lesions treated with NIPP [38]. Owing to the embryological origin of the vaginal and cervical epithelium being the same, the treatment strategy for VaIN and CIN is similar. Therefore, NIPP may be a treatment alternative for VaIN which needs randomized controlled trials to confirm.

While there is limited data on the adverse reactions following plasma coagulation in vaginal HSIL, the only two complications were pain requiring inpatient management and urinary retention in patients with vulvovaginal and perianal intraepithelial neoplasia [21]. Some physical treatments may lead to serious injuries, such as bladder damage [39] and sigmoid perforation with sepsis [40] associated with loop electrosurgical excision, or vaginal vault perforation requiring laparoscopic inspection associated with laser treatment [41]. However, when the argon beam penetrates through tissue, energy is lost rapidly, causing less damage to surrounding organs or deeper structures. Only mild local adverse reactions were recorded (burning sensation, pruritus, increased vaginal discharge, vaginal bleeding, and adhesion), without severe scarring or contracture until the end of follow-up in our study. Eight patients with multifocal lesions developed adhesions that could be easily separated with cotton swabs. As most patients develop complication of decrustation after physiotherapy, the main reaction in the APC group was vaginal drainage at 7–14 days after treatment, which was resolved at around 30–40 days.

## Conclusion

In conclusion, our results suggest that APC is safe and may result in an improved HPV clearance rate and cure rate, and a reduced recurrence rate, as compared to interferon therapy. Because APC has advantages such as ease of operation, short operation time, fast learning curve for surgeons, no intraoperative bleeding, no need for general anesthesia, and high safety, it may be a reasonable alternative to other physiotherapies for patients with VaIN I and VaIN II. However, a more robust, multicenter, and long-term follow-up RCT is recommended to validate our findings.

## Data Availability

The data that support the findings of this study are available on request from the corresponding author, LG, upon reasonable request.

## References

[1] Graham J, Meigs J (1952). Recurrence of tumor after total hysterectomy for carcinoma in situ. Am J Obstetr Gynecol.

[2] Kesic V, Carcopino X, Preti M, Vieira-Baptista P, Bevilacqua F, Bornstein J, Chargari C, Cruickshank M, Erzeneoglu E, Gallio N (2023). The European Society of Gynaecological Oncology (ESGO), the International Society for the Study of Vulvovaginal Disease (ISSVD), the European College for the Study of Vulval Disease (ECSVD), and the European Federation for Colposcopy (EFC) consensus statement on the management of vaginal intraepithelial neoplasia. Int J Gynecol Cancer.

[3] Sopracordevole F, Barbero M, Clemente N, Fallani M, Cattani P, Agarossi A, De Piero G, Parin A, Frega A, Boselli F (2016). High-grade vaginal intraepithelial neoplasia and risk of progression to vaginal cancer: a multicentre study of the Italian Society of Colposcopy and Cervico-Vaginal Pathology (SICPCV). Eur Rev Med Pharmacol Sci.

[4] Gunderson C, Nugent E, Elfrink S, Gold M, Moore K (2013). A contemporary analysis of epidemiology and management of vaginal intraepithelial neoplasia. Am J Obstet Gynecol.

[5] González Bosquet E, Torres A, Busquets M, Esteva C, Muñoz-Almagro C, Lailla J (2008). Prognostic factors for the development of vaginal intraepithelial neoplasia. Eur J Gynaecol Oncol.

[6] Wang L, Liu X, Zhang J, Li H, Wang X, Fu Y, Liu H, Xu Y, Meng L, Cui B (2023). Comparison of ALA-PDT and CO laser treatment of low-grade vaginal intraepithelial neoplasia with high-risk HPV infection: A non-randomized controlled pilot study. Photodiagn Photodyn Ther.

[7] Hu X, He Y, Lin L, Li X, Luo X, Wang L, Xu C (2023). 5-aminolevulinic acid photodynamic therapy combined with carbon dioxide laser therapy is a safe and effective treatment for vaginal intraepithelial neoplasia. Photodiagn Photodyn Ther.

[8] WHO classification of tumours of female reproductive organs, 4th edn. https://publications.iarc.fr/Book-And-Report-Series/Who-Classification-Of-Tumours/WHO-Classification

[9] Kim M, Lee I, Lee K (2018). Clinical outcomes and risk of recurrence among patients with vaginal intraepithelial neoplasia: a comprehensive analysis of 576 cases. J Gynecol Oncol.

[10] Hodeib M, Cohen J, Mehta S, Rimel B, Walsh C, Li A, Karlan B, Cass I (2016). Recurrence and risk of progression to lower genital tract malignancy in women with high grade VAIN. Gynecol Oncol.

[11] Freitas G, Costa A (2023). Non-Excisional therapeutic modalities in vaginal intraepithelial neoplasia. Eur J Obstet Gynecol Reprod Biol.

[12] Rountis A, Pergialiotis V, Tsetsa P, Rodolakis A, Haidopoulos D (2020). Management options for vaginal intraepithelial neoplasia. Int J Clin Pract.

[13] Inayama Y, Takamatsu S, Hamanishi J, Mizuno K, Horinouchi N, Yamanoi K, Taki M, Murakami R, Yamaguchi K, Kosaka K (2023). Imiquimod for cervical and vaginal intraepithelial neoplasia: a systematic review and meta-analysis. Obstet Gynecol.

[14] Li R, Hu Y, Wu M, Chen K (2022). Guttate psoriasis induced by interferon alfa-2b suppository treatment for high-grade cervical intraepithelial neoplasia. Dermatol Ther.

[15] Wang W, Liu Y, Pu Y, Li C, Zhou H, Wang Z (2021). Effectiveness of focused ultrasound for high risk human papillomavirus infection-related cervical lesions. Int J Hyperthermia.

[16] He M, Yu E, Hui S, Kung Y (2022). Clinical outcomes of laser vaporization for vaginal intraepithelial neoplasia—a 20-year retrospective review. Eur J Obstet Gynecol Reprod Biol.

[17] Prodromidou A, Pandraklakis A, Iavazzo C (2020). The emerging role of neutral argon plasma (PlasmaJet) in the treatment of advanced stage ovarian cancer: a systematic review. Surg Innov.

[18] Roman H, Auber M, Bourdel N, Martin C, Marpeau L, Puscasiu L (2013). Postoperative recurrence and fertility after endometrioma ablation using plasma energy: retrospective assessment of a 3-year experience. J Minim Invasive Gynecol.

[19] Grund K, Straub T, Farin G (1999). New haemostatic techniques: argon plasma coagulation. Bailliere’s Best Pract Res Clin Gastroenterol.

[20] Che C, Dong F, Wu X, Wang W, Jiang L (2019). Argon gas knife combined with cryotherapy for amyloidosis leading to severe airway stenosis. Respir Med Case Rep.

[21] Beavis A, Najjar O, Murdock T, Abing A, Fader A, Wethington S, Stone R, Ferriss J, Tanner E, Levinson K (2021). Treatment of vulvar and vaginal dysplasia: plasma energy ablation versus carbon dioxide laser ablation. Int J Gynecol Cancer.

[22] Wang N, Chai N, Li L, Li H, Zhai Y, Feng X, Liu S, Zhang W, Linghu E (2022). Comparison of endoscopic radiofrequency ablation and argon plasma coagulation in patients with gastric low-grade intraepithelial neoplasia: a large-scale retrospective study. Can J Gastroenterol Hepatol.

[23] Lee K, Park S, Shin E, Koh D, Lee J (2023). Argon plasma coagulation under direct peroral cholangioscopy for intraductal papillary mucinous neoplasm of the gallbladder. Endoscopy.

[24] Motz V, Lester C, Moyer M, Maranki J, Levenick J (2022). Hybrid argon plasma coagulation-assisted endoscopic mucosal resection for large sessile colon polyps to reduce local recurrence: a prospective pilot study. Endoscopy.

[25] Knabe M, Wetzka J, Welsch L, Richl J, Michael F, Blößer S, Heilani M, Kronsbein H, May A (2023). Radiofrequency ablation versus hybrid argon plasma coagulation in Barrett’s esophagus: a prospective randomised trial. Surg Endosc.

[26] Kushnir C, Fleury A, Hill M, Silver D, Spirtos N (2013). The use of argon beam coagulation in treating vulvar intraepithelial neoplasia III: a retrospective review. Gynecol Oncol.

[27] Ao M, Zheng D, Wang J, Gu X, Xi M (2022). A retrospective study of cytology and HPV genotypes results of 3229 vaginal intraepithelial neoplasia patients. J Med Virol.

[28] Li H, Guo Y, Zhang J, Qiao J, Geng L (2012). Risk factors for the development of vaginal intraepithelial neoplasia. Chin Med J.

[29] Chao A, Chen T, Hsueh C, Huang C, Yang J, Hsueh S, Huang H, Lin C, Tang Y, Liou J (2012). Human papillomavirus in vaginal intraepithelial neoplasia. Int J Cancer.

[30] Zeng H, Dai Q, Jiang D (2023). A single-institutional retrospective analysis of factors related to vaginal intraepithelial neoplasia. BMC Womens Health.

[31] Stuebs F, Dietl A, Koch M, Adler W, Geppert C, Hartmann A, Knöll A, Mehlhorn G, Beckmann M, Schulmeyer C (2023). Cytology and HPV co-testing for detection of vaginal intraepithelial neoplasia: a retrospective study. Cancers.

[32] Zhang Y, Xia R, Chen D, Zhang X (2022). Analysis of related factors of cervical intraepithelial neoplasia complicated with vaginal intraepithelial neoplasia. Clin Transl Oncol.

[33] Xiong Y, Cui L, Bian C, Zhao X, Wang X (2020). Clearance of human papillomavirus infection in patients with cervical intraepithelial neoplasia: a systemic review and meta-analysis. Medicine.

[34] Volcke A, Van Nieuwenhuysen E, Han S, Salihi R, Van Gorp T, Vergote I (2021). Experience with PlasmaJet™ in debulking surgery in 87 patients with advanced-stage ovarian cancer. J Surg Oncol.

[35] Wenzel T, Carvajal Berrio D, Reisenauer C, Layland S, Koch A, Wallwiener D, Brucker S, Schenke-Layland K, Brauchle E, Weiss M (2020). Trans-mucosal efficacy of non-thermal plasma treatment on cervical cancer tissue and human cervix uteri by a next generation electrosurgical argon plasma device. Cancers.

[36] Xiong J, Lin Z, He L, Zhang Y, Zeng G, Gui Q (2023). Analysis on the effect of radiofrequency ablation and electrocautery in the treatment of vaginal intraepithelial neoplasia. J Oncol.

[37] Marzi J, Stope M, Henes M, Koch A, Wenzel T, Holl M, Layland S, Neis F, Bösmüller H, Ruoff F (2022). Noninvasive physical plasma as innovative and tissue-preserving therapy for women positive for cervical intraepithelial neoplasia. Cancers.

[38] Weiss M, Arnholdt M, Hißnauer A, Fischer I, Schönfisch B, Andress J, Gerstner S, Dannehl D, Bösmüller H, Staebler A (2023). Tissue-preserving treatment with non-invasive physical plasma of cervical intraepithelial neoplasia-a prospective controlled clinical trial. Front Med.

[39] Indermaur M, Martino M, Fiorica J, Roberts W, Hoffman M (2005) Upper vaginectomy for the treatment of vaginal intraepithelial neoplasia. Am J Obstetr Gynecol 193(2):577–580; discussion 580–571. 10.1016/j.ajog.2005.03.055 10.1016/j.ajog.2005.03.055 16098901

[40] Powell J, Asbery D (2000). Treatment of vaginal dysplasia: just a simple loop electrosurgical excision procedure?. Am J Obstet Gynecol.

[41] Bogani G, Ditto A, Martinelli F, Mosca L, Chiappa V, Rossetti D, Leone Roberti Maggiore U, Sabatucci I, Lorusso D, Raspagliesi F (2018). LASER treatment for women with high-grade vaginal intraepithelial neoplasia: a propensity-matched analysis on the efficacy of ablative versus excisional procedures. Lasers Surg Med.

